# Histogram analysis parameters of apparent diffusion coefficient reflect tumor cellularity and proliferation activity in head and neck squamous cell carcinoma

**DOI:** 10.18632/oncotarget.25284

**Published:** 2018-05-04

**Authors:** Alexey Surov, Hans Jonas Meyer, Karsten Winter, Cindy Richter, Anna-Kathrin Hoehn

**Affiliations:** ^1^ Department of Diagnostic and Interventional Radiology, University Hospital of Leipzig, Leipzig 04103, Germany; ^2^ Institute of Anatomy, University Hospital of Leipzig, Leipzig 04103, Germany; ^3^ Department of Pathology, University Hospital of Leipzig, Leipzig 04103, Germany

**Keywords:** MRI, ADC, histogram analysis, KI 67, HNSCC

## Abstract

Our purpose was to analyze associations between apparent diffusion coefficient (ADC) histogram analysis parameters and histopathologicalfeatures in head and neck squamous cell carcinoma (HNSCC).

The study involved 32 patients with primary HNSCC. For every tumor, the following histogram analysis parameters were calculated: ADCmean, ADCmax, ADC_min_, ADC_median_, ADC_mode_, P10, P25, P75, P90, kurtosis, skewness, and entropy. Furthermore, proliferation index KI 67, cell count, total and average nucleic areas were estimated. Spearman's correlation coefficient (p) was used to analyze associations between investigated parameters.

In overall sample, all ADC values showed moderate inverse correlations with KI 67. All ADC values except ADCmax correlated inversely with tumor cellularity. Slightly correlations were identified between total/average nucleic area and ADC_mean_, ADC_min_, ADC_median_, and P25.

In G1/2 tumors, only ADCmode correlated well with Ki67. No statistically significant correlations between ADC parameters and cellularity were found.

In G3 tumors, Ki 67 correlated with all ADC parameters except ADCmode. Cell count correlated well with all ADC parameters except ADCmax. Total nucleic area correlated inversely with ADC_mean_, ADC_min_, ADC_median_, P25, and P90.

ADC histogram parameters reflect proliferation potential and cellularity in HNSCC. The associations between histopathology and imaging depend on tumor grading.

## INTRODUCTION

Head and neck squamous cell carcinoma (HNSCC) is one of the most frequent malignancies in humans [1]. Modern imaging modalities can not only correctly stage HNSCC but also provide additional information about tumor structure and behavior [1]. For instance, diffusion weighted imaging (DWI) by means of apparent diffusion coefficient (ADC) can predict tumor response to radiochemotherapy [2]. Furthermore, tumoral ADC values can be used for risk stratification of distant metastases [3]. In addition, ADC can reflect histopathological features of HNSCC, such as proliferation index and/or cellularity [4, 5].

Various ways to measure ADC values are described in the literature [6]. Usually, ADC is acquired by draw of a region of interest (ROI) in the largest slide of the tumor. In most reports, the mean value or ADC_mean_ within a ROI is estimated [6]. However, other ADC values can be also estimated by this approach: minimal ADC value or ADC_min_ and the maximal ADC value or ADC_max_ [6]. As reported previously, different ADC values might reflect different tissue compartments [6]. For instance, ADC_mean_ might represent the mean tissue composition and correlated with proliferation potential of investigated lesions [6, 7]. ADC_min_ has been reported to be associated with the part of tumors with most dense cellularity [6, 7].

A novel approach using every voxel of the ROI to issue a histogram of intensity levels could help to display histological features of tumors [8]. Using this method, the heterogeneity of tumor tissue might be better reflected than with single ADC values approach [8]. Besides ADC_mean_, ADC_min_, and ADC_max_ a broad spectrum of ADC parameters can be estimated: ADC percentiles, mode ADC, median ADC, kurtosis, skewness, and entropy [8]. These parameters display the first order statistical features of the measured ROI. The mode ADC represents the highest single value [8]. Kurtosis indicates the peakedness of the distribution, whereas skewness represents the asymmetry of the distribution [8]. Finally, entropy quantifies the irregularities of the distribution [8].

Previously, histogram analysis was performed to differentiate between histological tumor types [9–11], as a prognostic factor [12, 13] and as a predictive marker for therapy response [14]. Nevertheless, there are only few studies investigated correlations between parameters of ADC histogram analysis and the underlining histopathology in oncologic patients [15–18]. Recently, Shin *et al*. identified significant correlations between proliferation marker KI 67 and tumor cellularity with entropy and median ADC in breast cancer [15].

The purpose of this study was to analyze possible associations between ADC histogram analysis parameters and histopathological features in HNSCC.

## RESULTS

A complete overview of the estimated DWI parameters and histopathological findings is shown in Tables 1 and 2, respectively.

**Table 1:** ADC histogram analysis parameters of the investigated tumors

Parameters	Mean± standard deviation	Median	Range
**ADC_mean_**	1.14± 0.21	1.13	0.78–1.68
**ADC_min_**	0.70 ± 0.23	0.73	0.17–1.24
**ADC_max_**	1.78 ± 0.31	1.69	1.35–2.39
**P10**	0.90 ± 0.20	0.89	0.54–1.42
**P25**	1.00 ± 0.20	0.99	0.64–1.49
**P75**	1.27 ± 0.23	1.24	0.87–1.82
**P90**	1.42 ± 0.25	1.38	0.94–2.02
**Median**	1.11 ± 0.21	1.10	0.76–1.64
**Mode**	0.96 ± 0.27	0.97	0.18–1.55
**Standard deviation**	0.21 ± 0.006	0.21	0.08–0.4
**Kurtosis**	3.65 ± 1.36	2.98	2.23–7.93
**Skewness**	0.48 ± 0.47	0.48	−0.54–1.49
**Entropy**	2.45 ± 0.50	2.43	1.67-3.74

**Table 2:** Analyzed histopathological parameters

Parameters	Mean ± standard deviation	Median	Range
**Ki67**	64.56 ± 21.21	64	24–97
**Cell count**	196.1 ± 71.17	180	97–403
**Total nucleic area, μm^2^**	61 701.54 ± 26 867.95	57 262	24 971–161 797
**Average nucleic area, μm^2^**	353.64 ± 180.4	299	152–986

There were no significant differences of ADC parameters between G1/2 and G3 tumors (Table 3). Only entropy tended to be lower in G3 lesions (2.36 ± 0.52 vs 2.59 0.45, *P* = 0.08).

**Table 3:** Comparison of ADC histogram analysis parameters between G1/2 and G3 tumors

Parameters	G1/2 tumors	G3 tumors	*P* values
**ADC_mean_**	1.16 ± 0.14	1.13 ± 0.25	0.38
**ADC_min_**	0.74 ± 0.15	0.67 ± 0.28	0.47
**ADC_max_**	1.75 ± 0.25	1.80 ± 0.35	0.98
**P10**	0.93 ± 0.13	0.87 ± 0.24	0.18
**P25**	1.03 ± 0.13	0.97 ± 0.25	0.17
**P75**	1.27 ± 0.15	1.27 ± 0.28	0.65
**P90**	1.43 ± 0.19	1.41 ± 0.29	0.76
**Median**	1.14 ± 0.14	1.10 ± 0.25	0.27
**Mode**	1.01 ± 0.19	0.93 ± 0.32	0.30
**Standard deviation**	0.19 ± 0.005	0.21 ± 0.006	0.51
**Kurtosis**	3.39 ± 0.96	3.82 ± 1.58	0.59
**Skewness**	0.49 ± 0.31	0.48 ± 0.56	0.91
**Entropy**	2.59 ± 0.45	2.36 ± 0.52	0.08

In overall sample, all ADC values showed moderate inverse correlations with KI 67, ranged from –0.41 for ADC_mode_ to −0.58 for ADC_min_ (Table 4). Furthermore, all ADC values except ADC_max_ correlated inversely with tumor cellularity. The strongest correlation (–0.60) was identified for ADC_min_, P10, and P25. Also kurtosis correlated slightly with cell count (*p* = −0.37, *P* = 0.03). In addition, statistically significant correlations were identified between total nucleic area and ADC_mean_, ADC_min_, ADC_median_, P10, and P25 (Table 4). Also average nucleic area correlated moderately with ADC_mean_, ADC_min_, ADC_median_, P25, P75, and P90.

**Table 4:** Correlations between ADC histogram analysis and histopathological parameters in overall sample

Parameters	Ki67	Cell count	Total nucleic area	Average nucleic area
**ADC_mean_**	***p* = −0.54 *P* = 0.0014**	***p* = −0.56 *P* = 0.0009**	***p* = −0.35 *P* = 0.04**	***p* = −0.44 *P* = 0.01**
**ADC_min_**	***p* = −0.58 *P* = 0.0005**	***p* = −0.60 *P* = 0.0003**	***p* = −0.45 *P* = 0.009**	***p* = −0.42 *P* = 0.02**
**ADC_max_**	***p* = −0.46 *P* = 0.0079**	*p* = −0.03 *P* = 0.89	*p* = −0.10 *P* = 0.58	*p* = −0.35 *P* = 0.05
**P10**	***p* = −0.47 *P* = 0.0062**	***p* = −0.60 *P* = 0.0003**	***p* = −0.39 *P* = 0.03**	wp = −0.32 *P* = 0.07
**P25**	***p* = −0.52 *P* = 0.0022**	***p* = −0.60 *P* = 0.0003**	***p* = −0.45 *P* = 0.009**	***p* = −0.40 *P* = 0.03**
**P75**	***p* = −0.54 *P* = 0.0013**	***p* = −0.43 *P* = 0.01**	*p* = −0.27 P = 0.13	***p* = −0.47 *P* = 0.008**
**P90**	***p* = −0.45 *P* = 0.0092**	***p* = −0.47 *P* = 0.007**	*p* = −0.18 P = 0.32	***p* = −0.36 *P* = 0.04**
**Median**	***p* = −0.52 *P* = 0.0022**	***p* = −0.56 *P* = 0.0009**	***p* = −0.37 *P* = 0.03**	***p* = −0.45 *P* = 0.01**
**Mode**	***p* = −0.41 *P* = 0.02**	***p* = −0.51 *P* = 0.003**	*p* = −0.34 *P* = 0.059	*p* = −0.27 *P* = 0.14
**Standard deviation**	*p* = −0.16 P = 0.39	p = −0.009 P = 0.96	*p* = 0.10 *P* = 0.56	*p* = −0.12 *P* = 0.51
**Kurtosis**	*p* = 0.02 P = 0.93	***p* = 0.37 *P* = 0.03**	*p* = 0.10 *P* = 0.59	*p* = 0.18 *P* = 0.33
**Skewness**	*p* = −0.17 P = 0.34	*p* = 0.26 P = 0.15	*p* = 0.19 *P* = 0.31	*p* = 0.05 *P* = 0.79
**Entropy**	*p* = 0.11 P = 0.56	*p* = 0.14 P = 0.46	*p* = 0.11 *P* = 0.55	*p* = 0.15 *P* = 0.42

On the next step, separate correlation analysis for G1/2 and G3 tumors was performed. In G1/2 tumors, only ADC_mode_ correlated well with KI67 (*p* = –0.70, *P* = 0.0075). No other ADC parameters correlated statistically significant with proliferation index (Table 5). There were also no statistically significant correlations between ADC parameters and cellularity. Standard deviation of ADC values correlated well with average nucleic area (*p* = –0.59, *P* = 0.03) and total nucleic area (*p* = –0.72, *P* = 0.005).

**Table 5:** Correlations between ADC histogram analysis and histopathological parameters in G1/2 tumors

Parameters	Ki67	Cell count	Total nucleic area	Average nucleic area
**ADC_mean_**	*p* = −0.35 *P* = 0.24	*p* = −0.18 *P* = 0.55	*p* = 0.11 *P* = 0.72	*p* = 0.21 *P* = 0.48
**ADC_min_**	*p* = −0.45 *P* = 0.12	*p* = −0.43 *P* = 0.14	*p* = −0.19 *P* = 0.53	*p* = 0.04 *P* = 0.90
**ADC_max_**	*p* = −0.11 *P* = 0.72	*p* = 0.20 *P* = 0.51	*p* = 0.25 *P* = 0.40	*p* = 0.29 *P* = 0.34
**P10**	*p* = −0.53 *P* = 0.06	*p* = −0.23 *P* = 0.46	*p* = −0.19 *P* = 0.54	*p* = −0.03 *P* = 0.90
**P25**	*p* = −0.44 *P* = 0.13	*p* = −0.17 *P* = 0.58	*p* = −0.09 *P* = 0.76	*p* = 0.02 *P* = 0.96
**P75**	*p* = −0.24 *P* = 0.43	*p* = −0.08 *P* = 0.79	*p* = 0.13 *P* = 0.67	*p* = 0.26 *P* = 0.39
**P90**	*p* = 0.02 *P* = 0.96	*p* = −0.26 *P* = 0.39	*p* = 0.47 *P* = 0.10	***p* = −0.57** ***P* = 0.04**
**Median**	*p* = −0.34 *P* = 0.25	*p* = −0.15 *P* = 0.62	*p* = 0.05 *P* = 0.85	*p* = 0.16 *P* = 0.60
**Mode**	***p* = −0.70 *P* = 0.0075**	*p* = −0.11 *P* = 0.72	*p* = −0.13 *P* = 0.65	*p* = −0.10 *P* = 0.75
**Standard deviation**	p = 0.33 P = 0.28	p = −0.21 P = 0.48	***p* = −0.59 *P* = 0.03**	***p* = −0.72** ***P* = 0.005**
**Kurtosis**	*p* = −0.32 *P* = 0.29	*p* = 0.35 *P* = 0.25	*p* = −0.20 *P* = 0.51	*p* = −0.42 *P* = 0.15
**Skewness**	*p* = −0.30 *P* = 0.31	*p* = −0.09 *P* = 0.79	*p* = 0.05 *P* = 0.86	*p* = 0.07 *P* = 0.80
**Entropy**	*p* = −0.48 *P* = 0.10	*p* = 0.23 *P* = 0.46	*p* = 0.06 *P* = 0.83	*p* = 0.05 *P* = 0.87

In contrast to G1/2 lesions, G3 tumors showed multiple statistically significant associations between histopathological findings and imaging parameters (Table 6). Firstly, KI 67 correlated inversely with all ADC parameters except ADC_mode_ (Table 6). The identified correlations were stronger than in the overall sample and ranged from −0.62 for P10 to −0.69 for ADC_min_. Secondly, cell count correlated well inversely with all ADC parameters except ADC_max._ Strongest correlations were observed with P10 (*p* = −0.71), P25 (*p* = −0.72), and ADC_mode_ (*p* = −0.71). Also skewness showed a significant correlation with cellularity (*p* = 0.45, *P* = 0.04). Thirdly, total nucleic area correlated inversely with ADC_mean_, ADC_min_, ADC_median_, P25, and P90. No statistically significant correlations were found between ADC parameters and average nucleic area.

**Table 6:** Correlations between ADC histogram analysis and histopathological parameters in G3 tumors

Parameters	Ki67	Cell count	Total nucleic area	Average nucleic area
**ADC_mean_**	***p* = −0.68 *P* = 0.009**	***p* = −0.67 *P* = 0.001**	***p* = −0.46 *P* = 0.03**	*p* = 0.04 *P* = 0.87
**ADC_min_**	***p* = −0.69 *P* = 0.007**	***p* = −0.68 *P* = 0.009**	***p* = −0.49 *P* = 0.03**	*p* = 0.08 *P* = 0.97
**ADC_max_**	***p* = −0.64 *P* = 0.0026**	p = −0.17 P = 0.48	p = −0.32 P = 0.16	*p* = −0.13 *P* = 0.59
**P10**	***p* = −0.62 *P* = 0.0038**	***p* = −0.71 *P* = 0.005**	p = −0.42 P = 0.06	*p* = 0.16 *P* = 0.50
**P25**	***p* = −0.67 *P* = 0.001**	***p* = −0.72 *P* = 0.004**	***p* = −0.53 *P* = 0.02**	*p* = 0.04 *P* = 0.87
**P75**	***p* = −0.67 *P* = 0.001**	***p* = −0.56 *P* = 0.01**	p = −0.42 P = 0.06	*p* = 0.03 *P* = 0.99
**P90**	***p* = −0.66 *P* = 0.002**	***p* = −0.54 *P* = 0.01**	***p* = −0.45 *P* = 0.04**	*p* = −0.06 *P* = 0.80
**Median**	***p* = −0.68 *P* = 0.001**	***p* = −0.68 *P* = 0.001**	***p* = −0.48 *P* = 0.03**	*p* = 0.05 *P* = 0.84
**Mode**	*p* = −0.40 *P* = 0.08	***p* = −0.71 *P* = 0.005**	*p* = −0.37 *P* = 0.11	*p* = 0.27 *P* = 0.26
**Standard deviation**	*p* = −0.32 *P* = 0.17	*p* = 0.09 *P* = 0.72	*p* = −0.21 *P* = 0.38	*p* = −0.14 *P* = 0.55
**Kurtosis**	*p* = 0.20 *P* = 0.39	*p* = 0.34 *P* = 0.14	*p* = 0.27 *P* = 0.24	*p* = −0.02 *P* = 0.94
**Skewness**	*p* = −−0.11 *P* = 0.63	***p* = 0.45 *P* = 0.04**	*p* = 0.21 *P* = 0.38	*p* = −0.05 *P* = 0.85
**Entropy**	*p* = 0.26 *P* = 0.27	*p* = 0.24 *P* = 0.32	*p* = 0.37 *P* = 0.11	*p* = 0.39 *P* = 0.81

## DISCUSSION

This is the first report regarding relationships between parameters of ADC histogram analysis and clinically relevant histopathological findings in HNSCC to date.

Previously, only three studies analyzed associations between DWI and histopathology in HNSCC [4, 5, 19]. So, Driessen *et al.* correlated cell count, stroma area, nucleic and cytoplasmic areas, as well nucleic/cytoplasmic ratio with one DWI parameter, namely ADC_mean_ in 16 patients with laryngeal cancer [4]. Thereby, ADC_mean_ correlated inversely with cell count (*r* = −0.57, *P* = 0.02), nucleic area (*r* = –0.64, *P* = 0.03), and nucleic/cytoplasmic ratio (*r* = –0.77, *P* < 0.01). Furthermore, positive correlations were identified between ADC_mean_ and stroma area (*r* = 0.69, *P* = 0.01) [4]. In another study, three ADC values, namely ADC_min_, ADC_mean_, and ADC_max_ were correlated with cell count, KI 67, total and average nucleic areas in 11 patients with several primary HNSCC [5]. None of the ADC values correlated significantly with cell count [5]. Ki 67 correlated inversely with ADC_mean_ (*r* = –0.728, *p* = 0.011) and ADC_max_(*r* = –0.633, *p* = 0.036). In addition, also total nucleic area correlated well with ADC_mean_ (*r* = −0.691, *p* = 0.019) [5]. Finally, in the study of White *et al.* 18 patients with HNSCC were analyzed [19]. Tumor cellularity correlated significantly with ADC_mean_ (*r* = –0.556, *P* < 0.01). There were no significant correlations between ADC values and the percentages of stroma and necrosis [19].

Several factors may be causal for the controversial results of the reports. Firstly, as seen, there were investigations with very small number of patients. Secondly, the authors used different b values for ADC calculation and different ROI placement on ADC maps was performed. Thirdly, different scanners and Tesla strength were used.

In addition, in two reports one ADC parameter and in one study three ADC parameters were calculated. Presumably, they are non-sensible to detect all associations between imaging and histopathological features. Recently, some reports were published, which indicated that ADC histogram analysis parameters had a higher sensitivity in detection of associations with histopathological findings [8, 16–18]. For example, in thyroid cancer, it has been shown that ADC histogram analysis parameters can provide more detailed information on diffusion characteristics of tumors than commonly obtained ADC parameters [16]. Furthermore, in uterine cervical cancer, ADC histogram parameters were reported to be able to distinguish nodal positive from nodal negative tumors [17]. In addition, ADC entropy was identified as a potential imaging biomarker for tumor heterogeneity and p53 expression [17].

In the present study, 13 ADC histogram analysis parameters of HNSCC were calculated. They showed different significant correlations with several histopathological findings. In overall sample of 32 tumors, all ADC values showed inverse statistically significant moderate correlations with KI67 ranging from -0.41 (ADC_mode_) to -0.58 (ADC_min_). Thus, this finding suggests that ADC reflects proliferation potential of HNSCC and, therefore, can be used as surrogate marker of proliferation activity. Furthermore, our study documented significant inverse correlations between cell count and all ADC values except ADC_max_. Therefore, it can be postulated that ADC values can be used to assess tumor cellularity in HNSCC. As mentioned above, some authors indicated that other histopathological features, especially nucleic size and nucleic/cytoplasmic ratio influence water diffusion and ADC in HNSCC [4]. In the present study, however, only slightly-to-moderate correlations between total/average nucleic areas and different ADC values were found. Our findings let assume that ADC histogram analysis parameters cannot reflect nucleic characteristics in HNSCC or nucleic size/area play a low role in restriction of water diffusion.

Moreover, the present study identified another phenomenon. Our patients sample was heterogeneous and contained several HNSCC, i.e. well, moderately, and poorly differentiated tumors. We assumed that different lesions might show also different relationships between DWI parameters and histopathological findings. In fact, our results confirmed this hypothesis. Interestingly, in G1/2 tumors, only ADC mode correlated well with KI67. There were no significant correlations between the analyzed ADC parameters and tumor cellularity. This finding indicated that ADC histogram analysis cannot reflect tumor cell count in G1/2 HNSCC and, therefore, can also not been used as imaging marker for therapy control.

In contrast to well and moderately differentiated lesions, in G3 carcinomas, multiple statistically significant correlations between histopathological findings and ADC histogram analysis parameters were found. Especially, ADC_mean_, ADC_min_, P10, P25 and ADC_median_ showed best associations with cell count and KI 67, and total nucleic area. According to some previous studies, ADC_min_ has been reported to be best associated with cell count [20]. Our results showed that other parameters were just as well sensitive. Also in poorly differentiated tumors, skewness correlated significantly with cell count. Furthermore, several ADC parameters showed significant correlations with total nucleic areas. Overall, in high grade tumors, different ADC histogram analysis parameters reflect several histopathological features and can be used as imaging biomarker.

The exact cause of the fact that associations between ADC values and histopathology depended on tumor grading is unclear. Previously, only one study reported similar findings in meningiomas [21]. For instance, it has been shown that the association between ADC_min_ and cell count was stronger in grade II/III tumors (*r* = −0.79, *P* = .036) versus grade I meningiomas (*r* = −0.41, *P* = .008) [21]. Presumably, high grade tumors may have other relations between parenchyma and stroma than low grade lesion. Other factors, such as cell volume or vessel density may also play a role.

Always, independent of possible causes of the identified phenomenon, it is important to know that almost all ADC parameters reflect cellularity and proliferation potential in G3 HNSCC but not in G1/2 tumors. The knowledge of this finding can be helpful to optimize radiological control of therapy and design of further researches. Furthermore, it may also explain the mentioned above controversial results of the previous reports. Possibly, previous studies contained several proportions of G1, 2 and 3 tumors, which resulted in different associations between imaging findings and histopathology.

Our data suggest that ADC histogram analysis represent an important investigation method, which can really provide insight information regarding tissue composition in HNSCC. Furthermore, this method is more sensitive in comparison to positron emission tomography (PET) and dynamic contrast enhanced magnetic resonance imaging (DCE MRI). In fact, previous studies, which analyzed associations between PET and/or DCE MRI parameters and histopathological features in HNSCC, could find significant correlations between the variables. For example, it has been reported that PET parameters could not reflect cellularity and/or proliferation activity in HNSCC [5]. Similar results were also observed for perfusion parameters like volume transfer constant Ktrans and volume of the extravascular extracellular leakage space Ve [22].

The present study has several limitations. Although, it is larger than the previous reports, the number of acquired patients is relatively small. Furthermore, we analyzed only proliferation potential, cellularity and nucleic areas of the tumors. Other histopathological features, such as vascularity, invasiveness etc. were not investigated. There are aims for further researches.

In conclusion, our study identified the following: ADC histogram parameters reflect proliferation potential and cellularity in HNSCC. The associations between histopathology and imaging depend on tumor grading.

In G3 tumors, almost all ADC parameters correlated well with KI67 and cell count. Some ADC values showed statistically significant moderate correlations with total nucleic areas.

In low grade tumors, none of the ADC parameters correlated significantly with tumor cellularity. Only ADC mode correlated well with KI 67, and standard deviation of ADC values with total and average nucleic areas.

## MATERIALS AND METHODS

This prospective study was approved by the institutional review board and all patients gave their written informed consent.

### Patients

The study involved 32 patients with primary HNSCC, 8 (26%) women and 24 (74%) men, mean age of 56.5 ± 10.4 years, range 33–77 years. The localizations of the tumors were as follows: tonsil (*n* = 7, 21.9%), hypopharynx (*n* = 7, 21.9%), tongue (*n* = 7, 21.9%), oropharynx (*n* = 5, 15.6%), larynx (*n* = 5, 15.6%), and epipharynx (*n* = 1, 3.1%). In one patient (3,1%) well differentiated tumor (G1), in 12 patients (37.5%) moderately differentiated tumors (G2), and in 19 cases (59.4%) poorly differentiated carcinomas (G3) were diagnosed. The identified tumors were staged as T1 in one patient (3.1%), T2 in 7 patients (21.9%), T3 in 10 patients (31.2%), and as T4 in 14 cases (43.8%). Most patients (*n* = 29, 91.6%) had nodal metastases. Distant metastases occurred in 3 cases (9.4%).

### MR imaging

In all patients, neck MRI was performed using a combined head and neck coil. The imaging protocol included an axial T1 weighted (T1w) turbo spin echo (TSE) sequence prior and after intravenous application of contrast medium (Gadovist^®^, Bayer Healthcare, Leverkusen, Germany), with a dose of 0.1 mmol per kg of body weight, an axial T2 weighted (T2w) fat-supressed short tau inversion recovery (STIR) sequence, and an axial DWI EPI (echo planar imaging) sequence with b-values of 0 and 800 s/mm^2^ (TR/TE: 8620/73 ms, slice thickness: 4 mm, and voxel size: 3.2 × 2.6 × 4.0 mm).

### ADC histogram analysis

**Figure 1:**
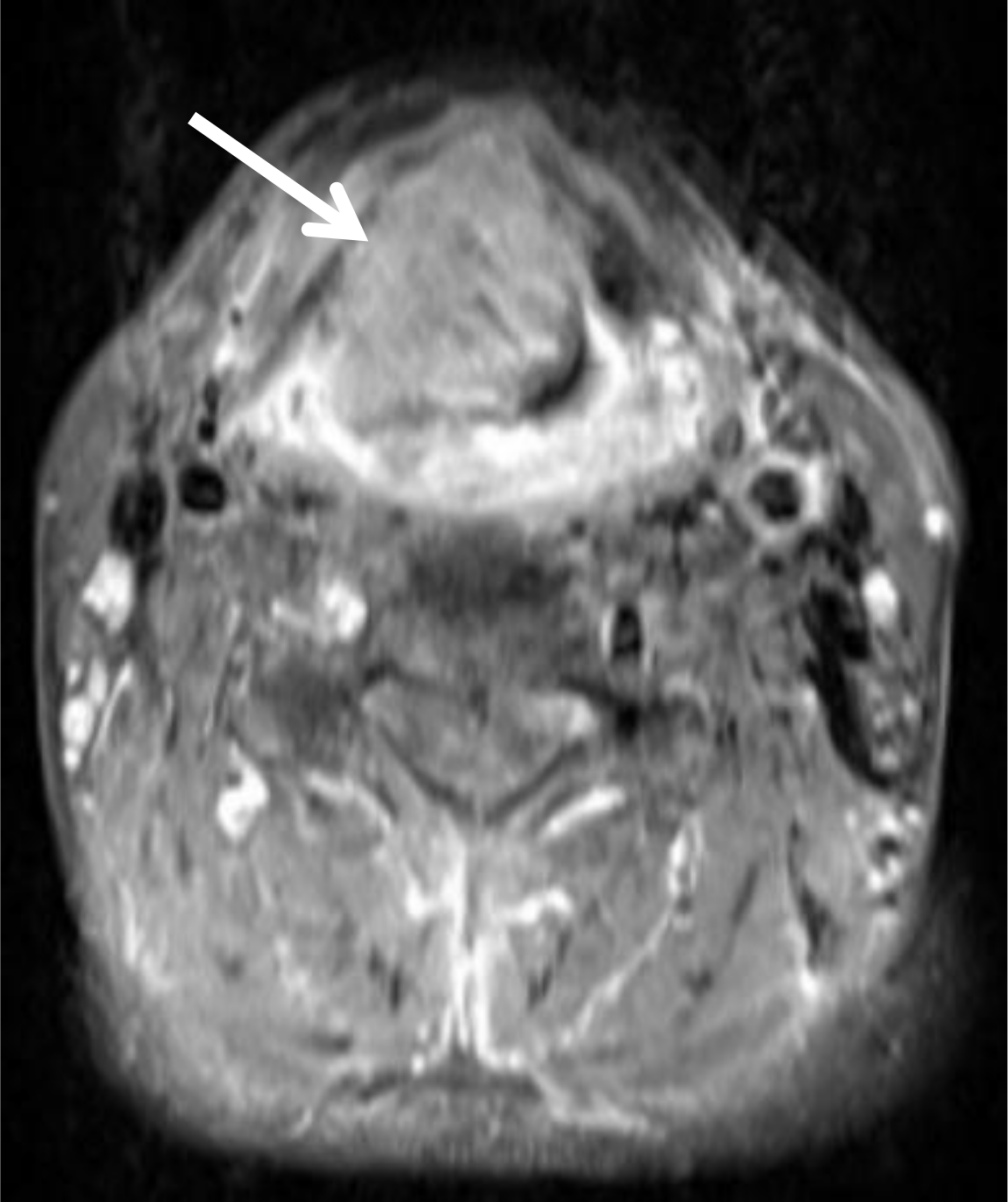

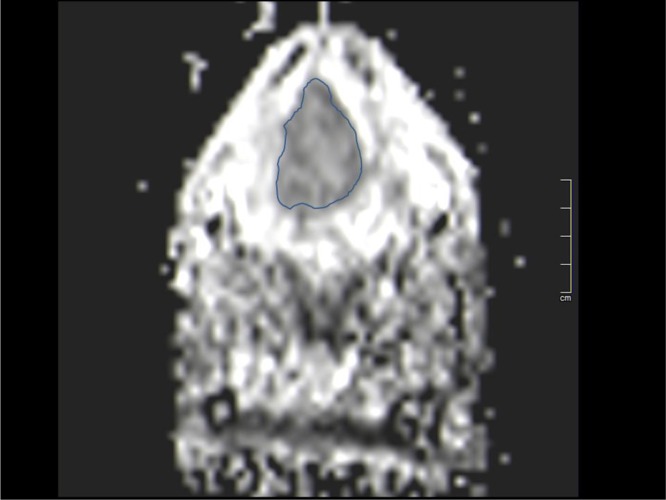

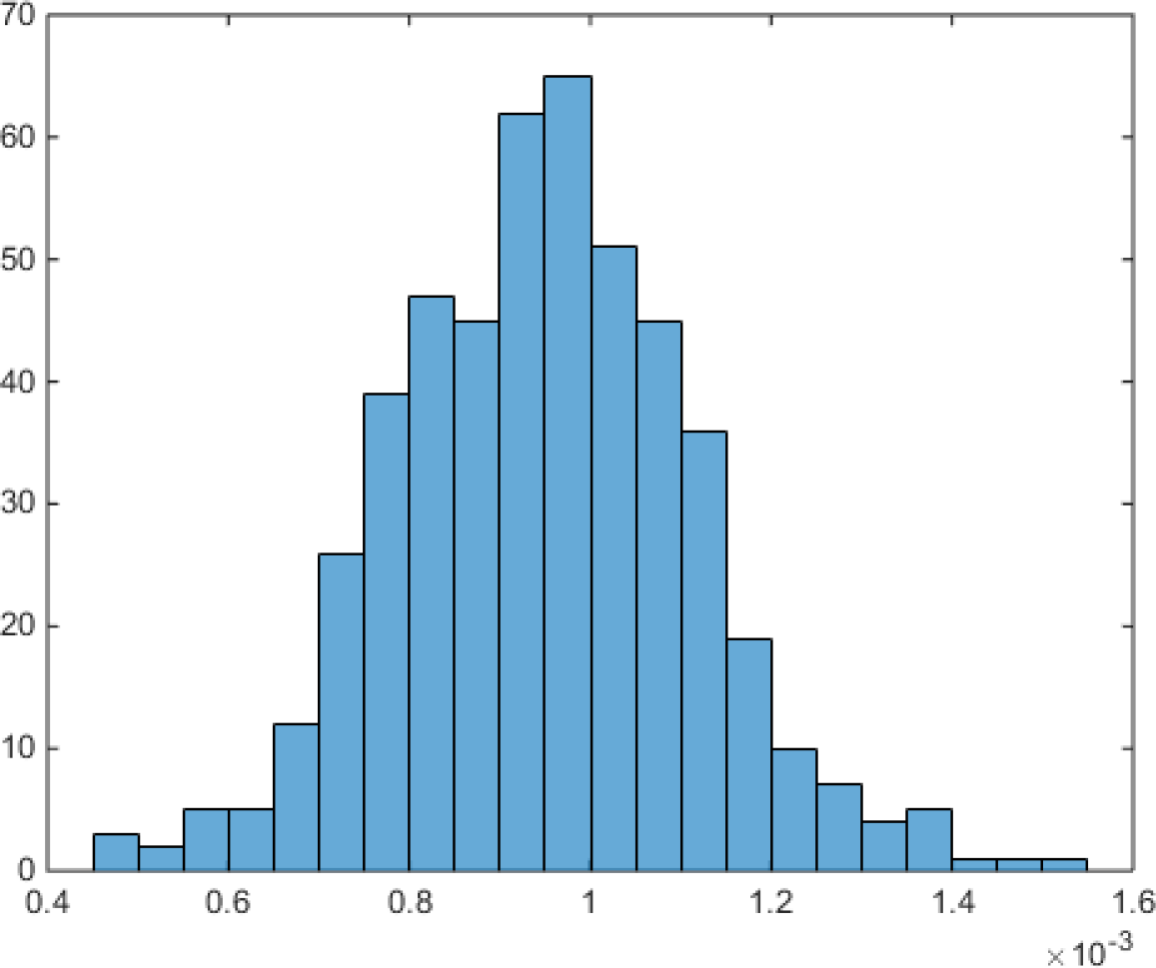

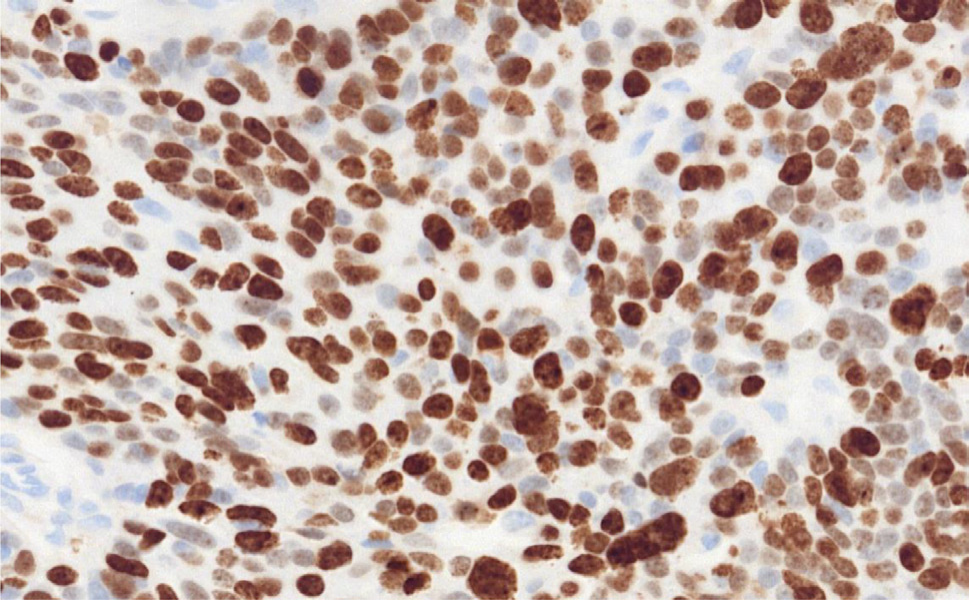
ADC histogram analysis parameters and histopathological findings in a large oral/oropharynx squamous cell carcinoma (**A**) T1 weighted image after intravenous administration of contrast medium showing a large oral lesion (arrow). (**B**) ADC map of the tumor with a ROI. (**C**) ADC histogram. The histogram analysis parameters (× 10^-3^ mm^2^s^-1^) are as follows: ADC_min_ = 0.45, ADC_mean_ = 0.95, ADC_max_ = 1.54, P10 = 0.74, P25 = 0.83, P75 = 1.05, P90 = 1.14, median = 0.95, mode = 0.98, and standard deviation = 0.17. Addionally, histogram-based characteristics are as follows: kurtosis = 3.43, skewness = 0.09, and entropy = 3.75. (**D**) Histopathological investigation after tumor biopsy. Immunohistochemical stain (MIB-1 monoclonal antibody). Ki 67 index is 86%, cell count is 190, total nucleic area = 58127 μm^2^, and average nucleic area = 310 μm^2^

For each tumor, automatically generated ADC maps were saved in DICOM format and processed offline with custom-made Matlab-based application (The Mathworks, Natick, MA) on a standard windows operated system. Polygonal regions of interest (ROI) were manually drawn on the transferred ADC maps along the contours of the primary tumor on each slice (whole lesion measure). All measures were performed by one radiologist (A.S., 15 years radiological experience). The position of every ROI was controlled on postcontrast T1 weighted images (Figure 1A and 1B). The following parameters were calculated: mean ADC (ADC_mean_), maximum ADC (ADC_max_), minimum ADC (ADC_min_), median ADC (ADC_median_), mode ADC (ADC_mode_). Furthermore, ADC percentiles: 10th (P10 ADC), 25th (P25 ADC), 75th (P75 ADC), and 90th (P90 ADC), as well histogram-based characteristics of the ROIs - kurtosis, skewness, and entropy – were estimated (Figure 1C) [23].

### Histopathological analysis

For this study, Ki 67 antigen stained specimens (MIB-1 monoclonal antibody, Dako Cytomation, Denmark) of the tumors were digitalized by using the Pannoramic microscope scanner (Pannoramic SCAN, 3DHISTECH Ltd., Budapest, Hungary) with Carl Zeiss objectives up to 41× bright field magnification by default. In the used bottom-up approach, the whole sample is acquired at high resolution. Low magnification representations are automatically obtained. Via Pannoramic Viewer 1.15.4 (open source software, 3D HISTECH Ltd., Budapest, Hungary) slides were evaluated and three captures with a magnification of ×200 were extracted of each sample. Further analyses of the digitalized histopathological images were performed by using the ImageJ software 1.48 v (National Institutes of Health Image program) with a Windows operating system [16–18].

Tumor proliferation index was estimated according the previous descriptions [16–18] as a ratio: (number of stained nuclei divided by number of all nuclei) ×100%. For the analysis, the area with the highest number of positive tumor nuclei was selected (Figure 1D).

Tumor cell count as a number of all nuclei, total nucleic area, and average nucleic area (total nucleic area/number of nuclei) were estimated as reported previously [16–18].

### Statistical analysis

Statistical analysis and graphics creation was performed using GraphPad Prism (GraphPad Software, La Jolla, CA, USA). Collected data were evaluated by means of descriptive statistics. Spearman's correlation coefficient (p) was used to analyze associations between investigated parameters. *P*-values < 0.05 were taken to indicate statistical significance.

## Abbreviations

MRI: magnetic resonance imaging
DWI: diffusion weighted imaging
ADC: apparent diffusion coefficient
HNSCC: head and neck squamous cell carcinoma
ROI: region of interest
